# A potential hypothesis for 2019-nCoV infection therapy through delivery of recombinant ACE2 by red blood cell-hitchhiking

**DOI:** 10.1186/s40709-020-00129-y

**Published:** 2020-12-07

**Authors:** Zahra Sadat Aghili, Seyed Abbas Mirzaei, Mehdi Banitalebi-Dehkordi

**Affiliations:** 1 Department of Molecular Medicine, School of Advanced Technologies, Shahrekord University of Medical Sciences, Shahrekord, Iran; 2 Department of Medical Biotechnology, School of Advanced Technologies, Shahrekord University of Medical Sciences, Shahrekord, Iran; 3 Cellular and Molecular Research Center, Basic Health Sciences Institute, Shahrekord University of Medical Sciences, Shahrekord, Iran

**Keywords:** ACE2, Drug delivery, Nanoparticles, 2019-nCoV, RBC hitchhiking

## Abstract

A novel infectious disease, caused by 2019 Novel Coronavirus (2019-nCoV) is responsible for the recent outbreak of severe respiratory disease. The 2019-nCoV spread rapidly and reaching epidemic proportions in many countries of the world. ACE2 was identified as a key receptor for 2019-nCoV infections. Excessive form of soluble ACE2 rescues cellular ACE2 activity which has a protective role in acute lung failure and neutralizes the virus. The short half-life of ACE2 is a major limitation to its practical application. Nanoparticle-based drug delivery systems are one of the most widely investigated approaches for developing novel therapies for a variety of diseases. Nevertheless, nanoparticles suffer from the rapid removal from the bloodstream by the reticuloendothelial system (RES). A noncovalent attachment of nanoparticles to RBCs increases their half-life in blood and allows transient accumulation in the lungs, while decreases their uptake by the liver and spleen. Connecting the recombinant ACE2 into the surface of nanoparticles that were attached to RBCs can be a potential therapeutic approach for 2019-nCoV infection through increasing their lung targeting to naturalize the virus and also acting as a bioreactor in the blood circulation to decrease serum level of Angiotensin II and protects lungs from injury/ARDS.

## Introduction

The 2019 Novel Coronavirus (2019-nCoV) or SARS-CoV-2 is an enveloped RNA virus that it is spreading rapidly and scientists are trying to discover drugs for an effective treatment [1]. This coronavirus induces excessive and abnormal non-effective host immune responses that are associated with acute respiratory distress syndrome (ARDS) and severe lung pathology, leading to death [2]. Spike (S) proteins of coronaviruses, including SARS-CoV and the 2019-nCoV, correlate with cellular receptors to mediate infection of their target cells [3]. It has been reported that the angiotensin-converting enzyme II (ACE2) plays an important role in the entry of the virus into cells, thus the ACE2-expressing cells may act as target cells and are susceptible to 2019-nCoV infection [4]. ACE2 is mainly expressed in alveolar epithelial type II cells. In most 2019-nCoV infected patients, lungs are heavily infected by the virus and alveolar epithelial cells serve as a viral reservoir [5]. ACE2 is also expressed in multiple extrapulmonary tissues including the human heart, kidneys, blood vessels, and intestine that might reveal the multiorgan dysfunction observed in patients [6, 7]. Researchers showed that 2019-nCoV directly infects and multiplies within the human blood vessel and kidney organoids [8]. Overexpression of human ACE2 enhanced disease severity in a mouse model of SARS-CoV infection, demonstrating that ACE2-dependent viral entry into cells is a critical step [9].

## Renin–angiotensin system in human coronavirus pathogenesis

There are two forms of ACE2. The full-length ACE2 is a type I integral membrane protein and the soluble form of ACE2 lacks the anchoring site and circulates in small amounts in the blood [10]; it has been shown to block the binding of the SARS-CoV spike protein to its receptor. ACE2 is a carboxypeptidase and is an ACE homologous. These receptors play critical functions in the renin-angiotensin system (RAS). ACE cleaves angiotensin I (Ang I) to form angiotensin II (Ang II); Ang II, the main active RAAS component, exerts its effects mainly via angiotensin II type 1 receptors (AT1R). Major effects of Ang II include vasoconstriction, renal sodium reabsorption and potassium excretion, aldosterone synthesis, blood pressure elevation, and induction of inflammatory and pro‐fibrotic pathways [11, 12], while, ACE2 negatively regulates the RAS and cleaves Ang II to formation of angiotensin 1–7 (Ang 1–7) which opposes the actions of Ang II [3, 12, 13]. Ang 1–7 exerts vasodilating, anti‐inflammatory, and anti‐fibrotic effects through binding to the Mas receptor [14]. Imai et al. showed that the RAS has a crucial role in severe acute lung injury and excessive ACE2 rescues cellular ACE2 activity which has a protective role in acute lung failure [13]. Indeed, Kuba et al. demonstrated that SARS CoV and the Spike protein of the SARS-CoV reduce ACE2 expression (but not ACE) in mice, contributing to severe lung failure [15].

## Recombinant ACE2 as a decoy receptor for 2019-nCoV

In vitro studies in monkey kidney cells, Vero E6, showed that a soluble form of ACE2 blocked association of the S1 domain with Vero E6 cells thus SARS-CoV replication was blocked [16]. Moreover, ACE2 fused to the Fc portion of immunoglobulin has just been described for high-affinity binding to the receptor-binding domain (RBD) of SARS-CoV and 2019-nCoV and potently neutralized SARS-CoV and 2019-nCoV in vitro [3]. Therefore, the rACE2 protein may be a potential therapeutic approach in the management of emerging lung disorders that suffer from ARDS [15, 17]. Nevertheless, the short half-life of rACE2 is the main limitation to its practical application [3].

## Nanoparticle-based targeted drug delivery

Successfully, in recent decades, nanoparticle-based drug delivery systems have been employed as experimentally and clinically to improve the efficacy of many drugs and therapeutic molecules. The field of nanomedicine provides distinct advantages over free drugs including targeting any organs while avoiding off-targets, and sustained release for improving the current treatment strategies for cancer and other diseases. But nanoparticles (NPs) suffer from the rapid clearance from the bloodstream by the reticuloendothelial system (RES) principally located in the liver and spleen, thereby limiting the dose available for the disease site. Due to this reason and other unfavorable factors, nanocarriers are not able to accumulate in many sites of therapeutic interest [18–20]. Erythrocytes are the particularly attractive vascular carrier for drug delivery of wide varieties of therapeutic agents to promote their biodistribution, pharmacokinetics, pharmacodynamics, and controlled release. RBC hitchhiking is a universal solution for dominant liver uptake and limited target organ deposition of nanocarriers as a drug delivery system. No damaging coupling to RBCs may prolong the lifetime of NPs in the circulation [21].

## Hypothesis and evaluation

Recently, it has been reported that the serum level of Ang II is significantly raised in 2019-nCoV infected patients and shows a linear positive correlation to viral load and lung injury [22]. Activation of the RAS causes extensive endothelial dysfunction and differing degrees of multiple organ (heart, kidney, and lung) injuries [23]. ACE2 has two different functions, both as the entry receptor of SARS-CoV causes to worsen the disease, and also as a negative regulator of the RAS, protect the lung from injury. Findings suggest that the soluble form of ACE2 decreases viral spread through competitively binding with 2019-nCoV and neutralizes the virus similar to a decoy receptor. In this way, it protects the lung from injury through saving cellular ACE2 activity and negatively regulates the RAS. Non-covalent attachments of NPs to the surface of RBCs (RBCs-NPs complex) increases their level in the blood without detectable changes in RBC circulation. This complex improves the blood pharmacokinetics and provides transient accumulation in the lungs while concurrently avoids their uptake by liver and spleen [18, 19, 24]. After several hours, NPs detach from the surface of RBCs, presumably due to shear force and interactions with vascular cells and ultimately are taken up by the liver and the spleen [24]. Recently, it has been reported that when RBCs are squeezed through the lung narrow capillaries, the NPs detach from the RBCs and transfer to the pulmonary capillary endothelial cells. Therefore, NPs, but not RBCs, accumulate transiently in the pulmonary vasculature [18, 19]. It can be a potential therapeutic approach for coronavirus infection. To use ACE2 as a treatment to 2019-nCoV-infected patients, we propose to fuse soluble form of recombinant ACE2 (rACE2) into the exposed surface of NPs that were attached to RBCs (therapeutic triple complex) and thereby we expect to achieve several results including:

- Increase the transient accumulation of rACE2 in highly vascularized organs like lungs, heart and kidneys.
- Extending the lifespan of the circulating rACE2.
- The therapeutic triple complex acts as a bioreactor in the blood circulation and decreases serum level of Ang II with cleavage it and inhibits severe acute lung injury/ARDS.

**Fig. 1:**
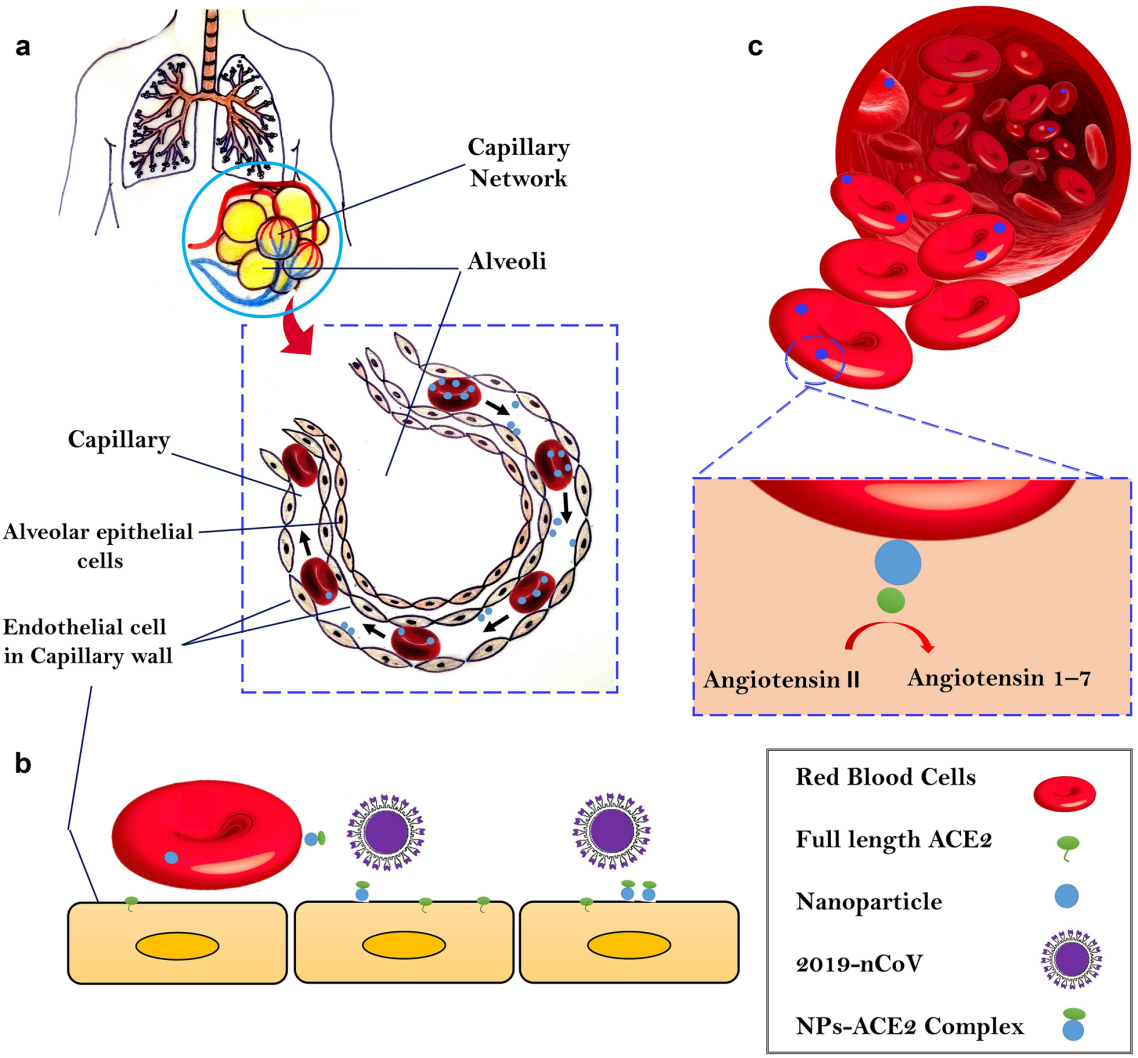
**a** Schematic of the therapeutic triple complex in blood circulation. **b** RBC–NPs complexes detach from RBCs in small capillaries, this process increases the rACE2 in lung capillaries and saving cellular rACE2 activity. **c** Also, it acts as a decoy for 2019-nCoV and decrease viral spreading. The therapeutic triple complex acts as a bioreactor in the blood circulation and decreases serum level of Ang II with cleavage it to Ang 1–7

For this purpose, at first NPs adsorb onto the RBCs ex vivo. Spherical (PSNP) NPs can readily adhere to RBCs membrane by incubation at particle/RBC ratios up to 100:1. Particle adhesion to RBCs membrane is likely due to electrostatic and hydrophobic interactions between PSNP and the RBC. After adsorption of NPs to RBCs, rACE2 passively will be adsorbed onto the exposed carboxylated surface of NPs. After injection via an intravascular catheter, the therapeutic triple complex augments the accumulation of rACE2 in highly vascularized organs, first of all, lungs. NPs attached to the rACE2, through the lung narrow capillaries, transfer from the RBCs to the pulmonary capillary endothelium under the shear force and interactions with vascular cells [18, 19] (Fig. 1).

## Effect of nanoparticles on biocompatibility of carrier RBCs

RBCs are naturally able to transport various cargoes throughout the circulatory system. RBC drug delivery systems represent viable (and, in some cases, preferable) alternatives to synthetic carriers; however, impairment of biocompatibility of the carrier RBCs and other adverse and unintended effects may cause other problems. Dosing, timing, and regimens of administration of carrier RBCs or targeted agents need to be assessed separately and adjusted for these new parameters of their behavior and influences in the body [25]. Attachment of NPs to the surface of carrier RBCs can lead to changes in circulation time, immunogenicity, biodistribution elimination pathways, and pharmacodynamics [26]. These changes in the pharmacokinetics of NPs imposed by carrier RBCs are beneficial for many drug delivery targets. To be clinically useful, NPs must not begin severe adverse effects on RBCs at loadings necessary for therapeutic applications [27]. In several studies, using two types of distinct NPs [PSNP and Lysozyme-dextran nanogels (LDNGs)], potential adverse and sensitizing effects of surface adsorption of NPs on the mouse and human RBCs were assessed. The seminal studies reported that RBCs may serve as “super-carriers” for NPs, as non-covalent attachment of PSNP to murine RBCs markedly alters the biodistribution of the NP in a manner advantageous to treatment of many diseases. PSNPs were used as a representative NP to evaluate RBCs as cellular carriers for NP delivery [27, 28]. According to these reports, the attachment of PSNP particle does not alter RBCs morphology [18, 19, 21, 24]. However, different studies have reported that the adsorption of PSNP beads onto RBCs causes their agglutination and sensitizes RBCs to damage by osmotic, mechanical and oxidative stress [27], whereas the adsorption of NPs made from soft biodegradable materials, LDNGs, does not induce negative effects on RBCs. Probable key physicochemical differences between LDNGs and PSNPs are likely responsible for the observed differences in their RBC sensitization and compatibility. LDNGs are less rigid, biodegradable and biocompatible compared to PSNPs. LDNGs do not induce NP-mediated damage to RBCs at loadings which PSNPs induce adverse effects [27, 28]. Therefore, the selection of the appropriate NPs types based on the targets of the experiments is an important factor and should be carefully studied.

## Conclusion

Recombinant ACE2 protein could not only be a treatment to block the spreading of 2019-nCoV, but modulation of the RAS could also be used to protect individuals with SARS. Fusing rACE2 into the exposed surface of NPs that were attached to RBCs, cause to extending the lifespan of the circulating rACE2 and increasing lung highly vascularized organs targeting and also act as a bioreactor in the blood circulation to decrease serum level of Ang II. Possibly this approach could also be used to protect individuals infected with other viruses such as SARS-CoV and avian influenza A strains, from developing acute severe lung failure and acute respiratory distress syndrome.

## Data Availability

Not applicable.

## Abbreviations

ACE: Angiotensin-converting enzyme
ACE2: Angiotensin-converting enzyme II
Ang: Angiotensin
ARDS: Acute respiratory distress syndrome
LDNGs: Lysozyme-dextran nanogels
2019-nCoV: 2019 Novel Coronavirus
NPs: Nanoparticles
PSNP: Polystyrene
rACE2: Recombinant ACE2
RAS: Renin-angiotensin system
RES: Reticuloendothelial system
RBCs: Red blood cells
